# Metagenomic Analysis of Human Diarrhea: Viral Detection and Discovery

**DOI:** 10.1371/journal.ppat.1000011

**Published:** 2008-02-29

**Authors:** Stacy R. Finkbeiner, Adam F. Allred, Phillip I. Tarr, Eileen J. Klein, Carl D. Kirkwood, David Wang

**Affiliations:** 1 Departments of Molecular Microbiology and Pathology & Immunology, Washington University School of Medicine, St. Louis, Missouri, United States of America; 2 Department of Pathology & Immunology, Washington University School of Medicine, St. Louis, Missouri, United States of America; 3 Department of Pediatrics, Washington University School of Medicine, St. Louis, Missouri, United States of America; 4 Department of Emergency Medicine, Children's Hospital and Regional Medical Center, Seattle, Washington, United States of America; 5 Enteric Virus Research Group, Murdoch Childrens Research Institute, Royal Children's Hospital, Victoria, Australia; The Pennsylvania State University, United States of America

## Abstract

Worldwide, approximately 1.8 million children die from diarrhea annually, and millions more suffer multiple episodes of nonfatal diarrhea. On average, in up to 40% of cases, no etiologic agent can be identified. The advent of metagenomic sequencing has enabled systematic and unbiased characterization of microbial populations; thus, metagenomic approaches have the potential to define the spectrum of viruses, including novel viruses, present in stool during episodes of acute diarrhea. The detection of novel or unexpected viruses would then enable investigations to assess whether these agents play a causal role in human diarrhea. In this study, we characterized the eukaryotic viral communities present in diarrhea specimens from 12 children by employing a strategy of “micro-mass sequencing” that entails minimal starting sample quantity (<100 mg stool), minimal sample purification, and limited sequencing (384 reads per sample). Using this methodology we detected known enteric viruses as well as multiple sequences from putatively novel viruses with only limited sequence similarity to viruses in GenBank.

## Author Summary

Diarrhea is one of the leading infectious causes of death worldwide with an estimated 1.8 million deaths annually, primarily in young children in developing countries. There are many known causes of diarrhea; however, the causes of ∼40% of the cases are still unknown. One possibility is that viruses that we currently do not know about are responsible for these cases. Thus, we used an experimental strategy termed “micro-mass sequencing” to systematically identify viruses present in stool from a number of patients suffering from diarrhea. Sequences from a number of novel viruses were detected, some which differed quite significantly from any previously described virus. These new viruses may or may not be responsible for causing diarrhea. Future studies will specifically address the potential of these viruses to cause human disease. One implication of this study is that there are likely to be many more unknown viruses that can be identified in this fashion. Furthermore, by studying these viruses, we will come to a more complete understanding of the role viruses play in diarrhea. Ultimately, this may lead to the development of therapeutics and/or vaccines that decrease the disease burden of diarrhea.

## Introduction

While traditional sequencing approaches are designed to characterize genomes of a single species of interest, metagenomic approaches, such as mass sequencing, transcend species boundaries allowing one to explore the makeup of microbial communities. Such methods provide a holistic look at microbial diversity within a given sample, completely bypassing the need for culturing [1]–[5]. Previous efforts in this field have explored the structure of virus communities in ecosystems as diverse as the ocean [1],[6] and the human gut [7],[8]. To date, the reported metagenomic studies of human stool have been limited to analysis of 4 specimens collected from 3 healthy patients [7],[8]. To our knowledge, no metagenomic investigation of the viral diversity found in human diarrhea has previously been described. Human diarrhea is the third leading cause of infectious deaths worldwide and is responsible for ∼1.8 million deaths in children under age five each year [9]. Bacteria, protozoa and viruses have all been implicated as causal agents. Chief among the known etiologic agents are rotaviruses, noroviruses, astroviruses, and adenoviruses [10] However, it is estimated that on average up to 40% of diarrhea cases are of unknown etiology, suggesting that unrecognized infectious agents, including viruses, remain to be discovered [11]–[15]. Mass sequencing affords an opportunity to explore the viral diversity (including both known and novel viruses) present in stool during acute episodes of diarrhea in a systematic and unbiased fashion, thereby laying the foundation for future studies aimed at assessing whether any novel or unexpected viruses detected play a causal role in human diarrhea.

In this study, mass sequencing was applied to explore specifically the viral communities present in pediatric patients suffering from diarrhea. We anticipated that the viral communities would vary significantly from specimen to specimen and that it would be desirable to sample broadly from multiple patients to obtain an overall perspective on the diversity of viruses that might be present. Toward this end, a simple yet robust experimental strategy was developed that circumvented certain technical and economic limitations of conventional mass sequencing. In both previous viral metagenomic studies of the human gut, large quantities of fecal matter (∼500 g) were collected from adults and then extensively purified to enrich for viral particles [7],[8]. In contrast, pediatric samples provide considerably smaller volumes of stool; therefore, our strategy was designed to minimize the number of physical purification steps so that as little as 30 mg of archived fecal matter could be analyzed. Here we present data generated by performing what we refer to as ‘micro-mass sequencing’ of several hundred sequence reads per sample from 12 different patients with acute diarrhea. This analysis provides evidence for the detection of known enteric viruses, viral co-infections, and novel viruses.

## Results

### Aggregate library analysis

Metagenomic analysis was carried out on fecal samples collected from 12 distinct pediatric patients suffering from acute diarrhea. Patient characteristics are shown in Table 1. A sequence independent PCR strategy was employed to amplify the extracted nucleic acids from each sample [16]. 384 clones were sequenced for each sample library. Because the goal of this project was to define the diversity of viruses present in the clinical specimens regardless of their relative abundance, nearly identical sequence reads were clustered to generate a set of non-redundant sequence reads. Unique, high quality sequence reads were then classified into broad taxonomic groups based on the taxonomy of the most frequent top scoring BLAST matches for each sequence. A total of 4,608 sequences were generated, of which 3,169 passed through a quality filter and 2,013 of those contained unique sequence information. Of the unique sequences passing through the filter, 1,457 (72%) could be identified by similarity to sequences in the Genbank nr database based on tBLASTx (E-value ≤10^−5^) alignments. The remaining 556 (28%) sequences had no significant similarity to any sequences in the nr database and were therefore categorized as being of ‘unknown’ origin. The 1,457 identifiable sequences were further classified into categories based on their proposed origin (Fig. S1). 519 (35.6%) were most similar to eukaryotic viruses, 25 (1.7%) to phage, 857 (58.8%) to bacteria, 3 (0.2%) to fungi, and 20 (1.4%) to human sequences. The remaining 33 (2.3%) were most similar to sequences that did not fall into the other previous categories and were consequently labeled as ‘other’. For example, some of the sequences had significant hits to mouse, fish, and plant genomes.

**Table 1:** Sample information.

Sample	Year Collected	Age of Patient	# of High Quality Sequence Reads	# of Unique Reads	Average Unique Read Length (bp)
D01	2005	14 mo	365	166	526
D02	1998	10 mo	193	87	536
D03	1984	NA	302	281	506
D04	1984	4 mo	311	154	626
D05	1980	NA	243	168	563
D06	2003	11 mo	153	132	393
D07	1999	23 mo	352	186	617
D08	1999	35 mo	302	167	255
D09	1981	NA	302	294	491
D10	1983	20 mo	195	146	447
D11	1978	NA	253	103	367
D12	2005	8 mo	198	129	300

### Individual library statistics

**Figure 1:**
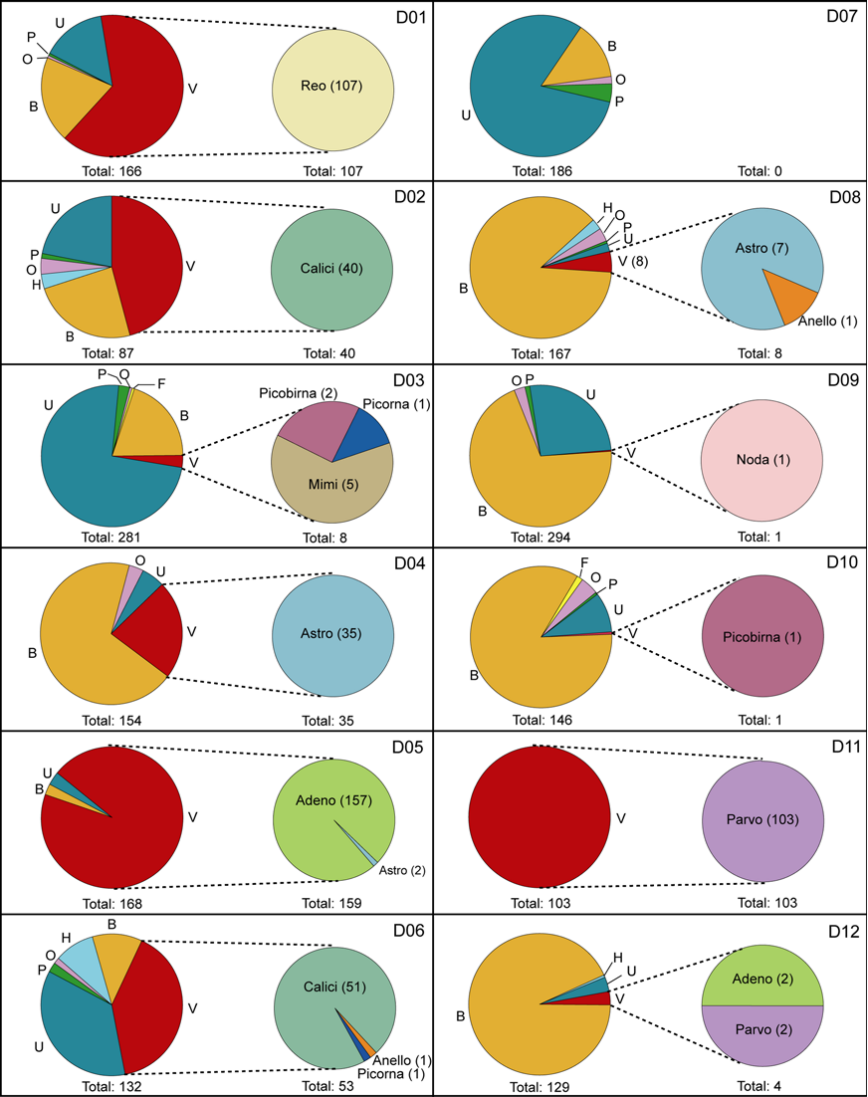
Categorization of sequence reads based on best tBLASTX scores (E-value: <10^−5^). Pies on the left side of each box depict the categorization of sequences from individual samples by phylotype: viral (V); phage (P); bacterial (B); human (H); fungal (F); other (O); and unassigned (U). Pies on the right side of each box depict further characterization of viral sequences by viral families/taxa: *Reoviridae* (Reo); *Caliciviridae* (Calici); *Astroviridae* (Astro); anellovirus (Anello); picobirnavirus (Picobirna); *Picornaviridae* (Picorna); mimivirus (Mimi); *Nodaviridae* (Noda); *Adenoviridae* (Adeno); *Parvoviridae* (Parvo). Numbers in parentheses indicate the number of sequence reads in each category.

384 clones were sequenced for each individual sample. The proportion of high quality sequences for each sample varied between 40% and 95% of the total clones (Table 1). The percentages of unique sequences per sample ranged from 41% to 97% of the high quality reads (Table 1). The average length of the unique, high quality sequences ranged from 255 to 626 bp. Viral sequences constituted between 0–100% of the reads in each library (Fig. 1). Some libraries (e.g., D01 and D05) were predominantly composed of viral sequences (64% and 95% respectively), whereas others consisted largely of bacterial (e.g., D08 and D12) or unassigned (e.g., D03 and D07) sequences. Based on the initial BLAST classification criteria, sequences with similarity to viruses from 7 different viral families and three unclassified genera (picobirnavirus, anellovirus and mimivirus) were detected in the 12 different samples (Fig. 1). Five of the samples (D03, D05, D06, D08, and D12) contained sequences from at least two different virus families known to infect humans.

### Detection of known viruses

The first specimen analyzed was a positive control stool specimen that had tested positive for rotavirus (D01) by enzyme immunoassay. It was our expectation that this sample would yield sequences derived from the infecting rotavirus. In this library, 107 non-redundant sequence reads were identified as viral in origin, almost all of which possessed ≥90% amino acid (aa) BLAST identity to known rotavirus sequences in Genbank. The sequence data included cloned fragments from all 11 RNA segments of the rotavirus genome.

An additional 11 stool specimens were then selected that had tested negative in conventional PCR and enzyme immunoassays for the known diarrhea viruses (rotaviruses, caliciviruses, astroviruses, and adenoviruses). Despite such screening, sequences derived from the canonical enteric viruses were detected in a number of samples. For example, calicivirus sequences were detected in D02 and D06, astrovirus sequences in D04, and adenoviruses were detected in D05 and D12. Almost all individual sequence reads in these cases possessed >90% aa identity to existing viral sequences in Genbank.

Adeno-associated virus (AAV), a member of the *Parvoviridae* family, was detected in two samples, D11 and D12. These viruses are known to infect the gastrointestinal tract, but are not thought to be enteric pathogens. For productive infections or reactivation from a latent state, AAV requires co-infection with a helper virus that is most commonly an adenovirus or less typically, a herpesvirus [17]. In D12, adenovirus sequences were detected. No additional viruses were detected in D11.

### Detection of novel virus sequences

**Figure 2:**
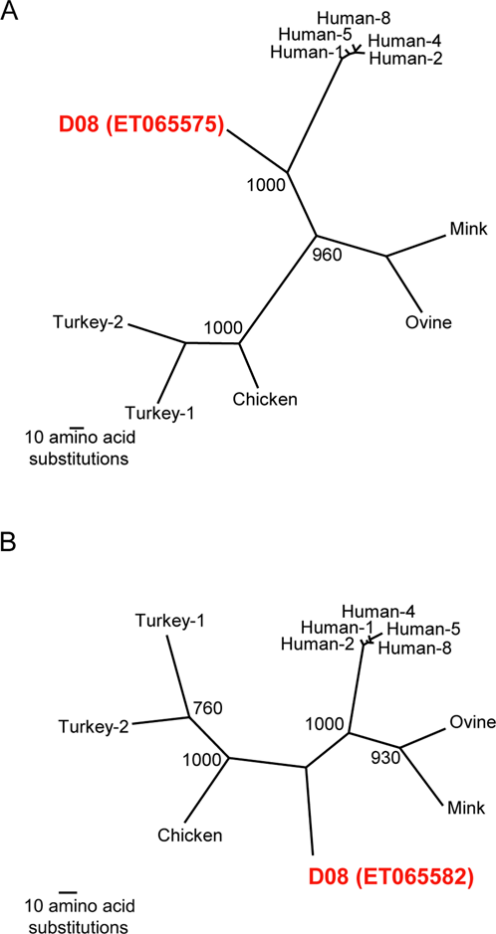
Phylogenetic analysis of highly divergent astrovirus-like sequence reads. Maximum parsimony phylogenetic trees were generated by comparing the translated amino acid sequence of individual sequence reads to the corresponding sequences from known astroviruses. 1,000 replicates were generated with bootstrap values over 700 shown. A) Representative sequence read mapping to astrovirus serine protease ORF (Accession number ET065575); B) Representative sequence read mapping to astrovirus RNA polymerase (Accession number ET065582).

**Figure 3:**
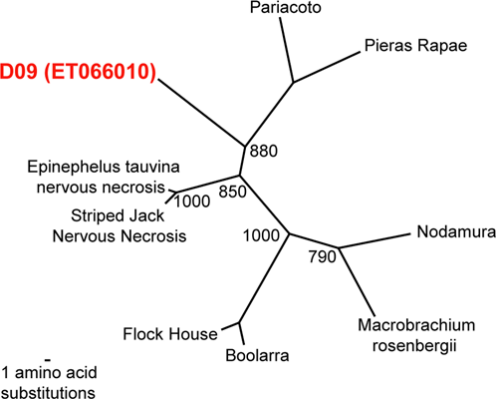
Phylogenetic analysis of a highly divergent nodavirus-like sequence read. Maximum parsimony phylogenetic trees were generated by comparing the translated amino acid sequence of one sequence read (Accession number ET066010) to the corresponding RNA polymerase sequences of nodaviruses. 1,000 replicates were generated with bootstrap values over 700 shown.

In many of the libraries, individual sequence reads were detected that possessed ≤ 90% aa identity to their highest scoring BLAST hit (representative sequences are listed in Table 2) suggesting that these sequences might be derived from novel viruses. In part because BLAST alignments are based on local sequence comparisons, BLAST is not an optimal method for making taxonomic assignments. In order to more accurately and precisely assess the relationship of these sequences to known viruses, we generated phylogenetic trees using the maximum parsimony method [18]. In cases where more than one sequence read hit the same region of a genome, only one representative sequence read is listed in Table 2 and phylogenetic trees are shown for only these representative sequences (Fig. 2–3 and Fig. S2, S3, S4, and S5). Phylogenetic analysis revealed that many of the sequences were divergent from known sequences on the order that approximated a distinct subtype or genotype (Fig. S2, S3, S4, and S5). This included two libraries with picobirnaviruses (D03, D10) (Fig. S2), two with picornaviruses (D03, D06) (Fig. S3), two with anelloviruses (D06, D08) (Fig. S4), and one with a norovirus (D06) (Fig. S5).

**Table 2:** Selected sequence reads with limited BLAST identity to known viruses.

Sample	Sequence Read Accession #	Identity to Top Hit	Top Hit (Accession #)	Virus Family/Taxa
D03	ET065742	78%	Human picobirnavirus strain 1-CHN-97 (AF246939)	Picobirnavirus
D03	ET065743	90%	Human coxsackievirus A19 (AF499641)	Picornaviridae
D06	ET067042	74%	Human enterovirus 91 (AY697476)	Picornaviridae
D06	ET067045	66%	TTV-like mini virus (AB026931)	Anellovirus
D06	ET067040	79%	Snow Mountain virus (AY134748.1)	Caliciviridae
D06	ET067041	88%	Norovirus C14 (AY845056.1)	Caliciviridae
D08	ET065575	57%	Human astrovirus 4 (AY720891)	Astroviridae
D08	ET065582	67%	Human astrovirus 5 (DQ028633)	Astroviridae
D08	ET065578	45%	TT virus (AB041963)	Anellovirus
D09	ET066010	35%	Epinephelus septemfasciatus nervous necrosis virus (AM085331)	Nodaviridae
D10	ET066456	81%	Human picobirnavirus 2-GA-91 (AF245701)	Picobirnavirus

In several instances, much more highly divergent sequences were detected that suggested that novel virus species might be present. The library generated for sample D08 included 7 unique sequence reads derived from two loci that displayed 52–67% aa identity to human astroviruses. Phylogenetic analysis of the individual sequence reads suggested that a novel astrovirus was present in D08 (Fig. 2). These sequence reads were assembled into two contigs, one of ∼800 bp that mapped to ORF1a and one of ∼500 bp that mapped to ORF1b. RT-PCR and subsequent sequencing of the amplicon confirmed the presence of the contigs in the original RNA extract as well as the contig assemblies (data not shown). Phylogenetic analysis of the two contigs yielded trees essentially identical to those generated from the individual sequence reads (data not shown).

In sample D09, we detected one sequence read which exhibited limited similarity to viruses in the family *Nodaviridae* (Table 2). RT-PCR of this sample using primers designed from the sequence read confirmed the presence of a 229 bp fragment in the original RNA extract (data not shown). Phylogenetic analysis of the sequence of the RT-PCR product demonstrated that the nodavirus in sample D09 was highly divergent from other known nodaviruses (Fig. 3).

Finally, one sample, D03, contained five sequence reads that, based on the top tBLASTX hits, contained 47% to 52% aa identity to endonuclease genes in the amoeba-infecting virus *Acanthamoeba polyphaga* mimivirus. These sequences also possessed approximately similar levels of sequence identity to a number of bacterial genomes and phage genomes containing putative endonuclease proteins. Phylogenetic analysis comparing the sequence reads to the top scoring BLAST hits (Fig. S6) did not conclusively clarify the origin of these sequences. Further experimentation will be required to unambiguously determine if these sequences are derived from a mimi-like virus, phage, or a bacterial species.

### Unassigned sequences

Some sequences in the libraries had no significant hits to any sequences in the Genbank nr database. Samples D03 and D07 had a large abundance of these ‘unassigned’ reads. Relaxing E-value thresholds for designating various sequence categories resulted in the ability to classify a greater number of these unassigned sequences; however, many of these classifications likely represent artifactual alignments. Viral assignments remained largely unaffected, even when E-value thresholds as permissive as 10 were applied.

## Discussion

We examined the diversity of viral communities in stools from 12 children with diarrhea using a strategy we describe as ‘micro-mass sequencing’. This strategy, which entails crude purification of fecal suspensions, nucleic acid purification, random PCR amplification, and cloning and sequencing of several hundred colonies, effectively detected known enteric viruses, viral co-infections, and novel viruses. In most traditional metagenomic studies, large sample volumes are subjected to multiple stages of filtration and purification before sequencing. For example, in previous metagenomic studies of the gut, 500 g of fecal samples were initially collected for the analyses. Because clinical pediatric diarrhea specimens are much more limited in volume, we chose to both minimally purify the samples and to employ a random PCR amplification strategy. These combined steps enabled us to rapidly generate sequencing libraries from small quantities of archived stools (30–100 mg). Furthermore, we wished to sample broadly from multiple patients because of the large number of viruses known or suspected to be associated with diarrhea. Therefore, rather than sequence few specimens in great depth as has been done previously (10,000 sequences per sample) [8], we focused on sequencing fewer clones (384 per sample) from more samples (12 specimens).

Our analysis detected viruses, bacteria, host, phage and other sequences (Fig. 1). The presence of non-viral sequences in the libraries was not surprising as only minimal efforts were made to enrich for viral sequences. In fact, the goal of this strategy was to manipulate the specimens as little as possible in the interest of simplicity. Even so, in a few libraries, 100% of the sequence reads were of viral origin. Additional processing, such as treating the specimens with DNase, reduced the background signal and increased the percentage of viral reads in some instances (data not shown).

Viral sequences were detected in all but one sample. Interestingly, a number of DNA viruses (bacteriophages, adenoviruses, and adeno-associated viruses) were detected in our analysis, despite our use of a methodology focused on purification of RNA. While it is possible that RNA transcripts from these viruses were purified [19], it is more likely that viral DNA was co-purified with RNA, as is common in other RNA purification methods [20]. PCR analysis of samples D05 and D11 in the absence of reverse transcription, yielded positive results for adenovirus and adeno-associated virus, respectively, indicating that viral DNA was present in the RNA preparations (data not shown).

Analysis of this initial cohort of 12 specimens yielded a wealth of original findings. In contrast to previous metagenomic studies of stool [8], a number of known human viruses were detected in these clinical specimens. These included common enteric pathogens such as rotavirus, adenovirus, calicivirus, and astrovirus. In addition, putatively benign adeno-associated viruses (AAV) were also detected which are not generally associated with human diarrhea. Aside from one sample known to contain rotavirus, we intended to analyze the viral communities present in samples that were not infected by known enteric pathogens in order to identify viruses that might be responsible for the unexplained cases of diarrhea. The fact that micro-mass sequencing detected these canonical viruses in some of the specimens, despite conventional diagnostic testing by EIA and PCR, underscores the sensitivity limits of conventional diagnostics.

### Detection of novel viruses

Sequences were detected in this study from at least 9 putatively novel viruses. For 7 of these sequences, the degree of divergence observed based on phylogenetic analysis suggested that they might represent novel virus subtypes or genotypes of picobirnavirus, enterovirus, TT virus and norovirus (Fig. S2, S3, S4, and S5). Picobirnaviruses belong to an unclassified genus of double stranded RNA viruses and have been detected in fecal matter from human and other animals both with and without diarrhea [21]. Only a limited number of picobirnavirus sequences have been previously described in the literature and thus the identification of two novel picobirnaviruses significantly expands the known diversity of this taxonomic group, underscoring the unrecognized viral diversity inhabiting the human body.

Sequences representing a divergent norovirus were detected in sample D06 (Fig. S5). Phylogenetic analysis of individual sequence reads that mapped to the RNA polymerase and the NS4 regions of human norovirus suggested that these sequences were derived from a novel or unsequenced member of norovirus genogroup 2. In the initial screening by conventional PCR, this sample tested negative for norovirus. Upon closer examination, four mutations were observed in one of the PCR primer binding sites, which plausibly hindered the PCR screening assay [14].

In two samples, much more highly divergent sequences were detected. In D08, phylogenetic analysis of 7 unique sequence reads strongly suggested that a novel astrovirus species was present (Fig. 2). The observed sequence variation between these sequence reads and the known astrovirus genomes greatly exceeds the variation that exists between the 8 known serotypes of human astrovirus, suggesting that this virus is not simply another serotype of the known astroviruses. Astroviruses are non-enveloped, single stranded, positive sense RNA viruses that account for up to 10% of sporadic diarrhea cases [22]. Infections with astroviruses most frequently cause watery diarrhea lasting 2–4 days, and, less commonly vomiting, headache, fever, abdominal pain, and anorexia in children under the age of 2, the elderly, and immunocompromised individuals [23]. The detection of this genetically distinct astrovirus raises the question as to whether or not this is an authentic human virus, and if so, whether or not it is a causal agent of human diarrhea.

Another novel sequence detected appeared by phylogenetic analysis to belong to the family *Nodaviridae*. Nodaviruses are small single-stranded, positive sense, bipartite RNA viruses, divided into two genera, the alphanodaviruses (insect viruses) and the betanodaviruses (fish viruses). Currently, none of the established family members are known to naturally infect mammals although experimental manipulation of the viral genome has enabled viral replication in a wide array of organisms including mammals [24]. While it is tempting to speculate that this might represent the first instance of human infection with a nodavirus, further experimentation such as serological analysis is required to definitively answer this question. Another plausible explanation is that the virus may be present simply as a result of consumption of fish infected by the virus. A prior report describing the presence of plant virus RNAs in human stool has similarly been attributed to dietary exposure [8]. Incidentally, some fish genomic sequences were detected in this particular sequence library (D09 “other” bin) supporting the possibility of dietary exposure. However, the potential piscine origin of this virus would not necessarily preclude its role as an etiologic agent of human disease.

The micro-mass sequencing approach, like any other experimental methodology capable of detecting novel viruses (such as culture or degenerate PCR), cannot of course by itself determine whether the newly detected agent is pathogenic. However, this strategy can generate novel, testable hypotheses such as “Are these novel viruses involved in the etiology of human diarrhea?” and “What is the true host of these viruses?” that could not be asked in the absence of the knowledge that these viruses existed.

### Unassigned reads

556 out of the 2013 (28%) unique high quality sequences were binned as unassigned by the BLAST criteria. Of these, 23 were identified as containing repetitive elements or low-complexity sequence by RepeatMasker [25],[26] thus explaining the lack of meaningful BLAST alignments. The origin of the remaining 533 sequences that were unassigned is uncertain, but they could be derived from unannotated host genome, novel or unsequenced microbes, or dietary sources which have not been sequenced. However, it is also possible that some of these sequences could represent viruses that have no appreciable similarity to sequences of currently known viruses. Extracting more telling information from these sequences is a challenging problem that will require the development of new computational measures capable of detecting more distant evolutionary relationships than is possible with existing methods. In addition, as more genome sequences from diverse organisms and other genomic/metagenomic projects become available, sequence similarity based methods may identify a greater fraction of these currently unassigned sequences.

### Diagnostic applications and implications

Our data suggest that micro-mass sequencing might be of great diagnostic utility for a number of reasons. First, viruses escaping detection in conventional assays were detected by micro-mass sequencing. In theory, the sensitivity of this strategy is limited only by the depth of sequencing. As demonstrated here, even shallow sequencing performed better than conventional diagnostics in some instances. In addition, the unbiased nature of the method enabled detection of viruses not conventionally tested for. Moreover, co-infections were detected in multiple samples. Furthermore, for multi-segmented viruses such as rotaviruses, reassortment of segments between species is a major mechanism of viral evolution that can lead to the emergence of more virulent strains [27]. Complete genome sequencing of all segments simultaneously would yield completely unambiguous identification of the viral genotype. In contrast to typical PCR or antibody based assays that target a single segment or protein, micro mass sequencing detected all 11 genomic RNA segments of rotavirus. In terms of technical practicality, samples were only minimally manipulated relative to traditional metagenomic sequencing [1],[3],[6],[8], thereby avoiding the time, labor, and use of specialized equipment required to concentrate the specimens, rendering this methodology potentially amenable to use in diagnostic laboratories. As sequencing costs diminish and efficiencies improve, mass sequencing could become a powerful diagnostic tool.

In summary, we have shown that micro-mass sequencing can define the diversity of viral communities found in fecal samples from diarrhea patients. Both known viruses and novel viruses were detected by sequencing only a few hundred colonies from each sample library. These studies will serve as the springboard for further interrogations of the roles of these diverse viruses in the gastrointestinal tract. Finally, our detection of multiple novel viruses in this initial, limited exploration of a dozen samples suggests that broader sampling of patient specimens is likely to be highly fruitful in terms of identification of additional novel viruses.

## Materials and Methods

### Clinical archived stool specimens

#### Melbourne Cohort

Stool samples were collected from children under the age of 5 who were admitted to the Royal Children's Hospital, Melbourne, Victoria, Australia with acute diarrhea between 1978 and 1999.

#### Seattle Cohort

Stool samples were collected between 2003–2005 at the Emergency Department of the Children's Hospital and Regional Medical Center in Seattle, Washington, USA as part of a prospective study attempting to discern the cause of unexplained pediatric diarrhea.

### Diagnostic testing of stool specimens for known microbial diarrheagenic agents

#### Melbourne Cohort

Specimens were tested by routine enzyme immunoassays (EIA) and culture assays for rotaviruses, adenoviruses, and common bacterial and parasitic pathogens as previously described [14]. RT-PCR assays were used to screen specimens for the presence of caliciviruses and astroviruses [14],[28].

#### Seattle Cohort

Specimens were tested for the presence of a number of bacterial species (*Campylobacter jejuni*, *Escherichia coli* O157∶H7 and non-O157∶H7 Shiga toxin-producing *E. coli*, *Salmonella*, *Shigella*, and *Yersinia*) following standard culture assays, *Clostridium difficile* toxin by a cytotoxicity assay, parasites by microscopy and antigen testing [29]. Additionally, samples were tested by EIA for rotaviruses, adenoviruses, noroviruses 1 & 2, and astroviruses (Meridian Biosciences, DAKO). This study was approved by the institutional review boards of the CHRMC and of Washington University.

### Library construction and mass sequencing

Chips of frozen archived fecal specimens (∼30–150 mg) were resuspended in 6 volumes of PBS. A subset of the archived specimens had been previously diluted and were further diluted 1∶1 in PBS. The stool suspensions were centrifuged (9,700×g, 10 minutes) and supernatants were harvested and then passed through 0.45 µm filters. RNA was extracted from 100 µL of the filtrates using RNA-Bee (Tel Test, Inc., Friendswood, Texas) according to manufacturers instructions. Approximately, 100–300 nanograms of RNA from each sample was randomly amplified following the Round AB protocol as previously described [16]. The amplified nucleic acid was cloned into pCR4 using the TOPO cloning kit (Invitrogen, Carlsbad, CA), and transformed into Top10 bacteria. Positive colonies were subcloned into 384 well plates, DNA was purified using magnetic bead isolation, and followed by sequencing using standard Big Dye terminator (v3.1) sequencing chemistry and the universal primer M13 reverse. Reactions were ethanol precipitated and resuspended in 25 uL of water prior to loading onto the ABI 3730xl sequencer.

### Analysis of sequence reads

Sequence traces were subjected to quality assessment and base-calling using Phred [30],[31]. Lucy [32] was used to trim vector and low quality sequences. Default parameters were used except that high quality sequences identified by Lucy were allowed to be as short as 75 nucleotides. To define the set of reads with unique sequence content in each library, sequences that passed the quality filter were clustered using BLASTClust from the 2.2.15 version of NCBI BLAST to eliminate redundancy. Sequences were clustered based on 98% identity over 98% sequence length, and the longest sequence from each cluster was aligned to the NCBI nr database using the tBLASTx algorithm [33]. An E-value cutoff of 1e-5 was applied. Sequences were phylotyped as human, bacterial, phage, viral, or other based on the identity of the best BLAST hit. Sequences without any hits having an E-value of 1e-5 or better were placed in the “Unassigned” category. All eukaryotic viral sequences were further classified into viral families in similar fashion. Trimmed, high quality sequences that were not found by RepeatMasker to contain repetitive or low-complexity sequence have been deposited in Genbank (Accession numbers ET065304 through ET067293).

### Phylogenetic analysis

ClustalX (1.83) was used to perform multiple sequence alignments of the protein sequences associated with select sequence reads. Available nucleotide or protein sequences from known viruses were obtained from Genbank for inclusion in the phylogenetic trees. Selected sequences from Genbank included those with the greatest similarity to the sequence read in question based on the BLAST alignments as well as representative sequences from all major taxa within the relevant virus family. The protein alignments created by ClustalX were input into PAUP [18], and maximum parsimony analysis was performed using the default settings with 1,000 replicates.

Astrovirus trees: Human astrovirus 1 (NC_001943); Human astrovirus 2 (L13745); Human astrovirus 3 (AAD17224); Human astrovirus 4 (DQ070852); Human astrovirus 5 (DQ028633); Human astrovirus 6 (CAA86616); Human astrovirus 7 (AAK31913); Human astrovirus 8 (AF260508); Turkey astrovirus 1 (Y15936); Turkey astrovirus 2 (NC_005790); Turkey astrovirus 3 (AY769616); Chicken astrovirus (NC_003790); Ovine astrovirus (NC_002469); and Mink astrovirus (NC_004579).

Nodavirus tree: Striped Jack Nervous Necrosis virus (Q9QAZ8); Macrobrachium rosenbergii nodavirus (Q6XNL5); Black Beetle virus (YP_053043.1); Flockhouse virus (NP_689444.1); Epinephelus tauvina nervous necrosis virus (NC_004136.1); Nodamura virus (NC_002691.1); Boolarra virus (NC_004145.1); Pariacoto virus (NC_003692.1); and Redspotted grouper nervous necrosis virus (NC_008041.1).

Picornavirus trees: Human coxsackievirus A1 (AAQ02675.1), Human coxsackievirus A18 (AAQ04836.1), Human coxsackievirus A19 (AAQ02681.1), Human coxsackievirus A21 (AAQ04838.1), Human coxsackievirus A24 (ABD97876.1), Human poliovirus 1 (CAD23059.1), Human coxsackievirus A2 (AAR38840.1), Human coxsackievirus A4 (AAR38842.1), Human coxsackievirus A5 (AAR38843.1), Human coxsackievirus A16 (AAV70120.1), Human enterovirus 89 (AAW30683.1), Human enterovirus 91 (AAW30700.1), Human enterovirus 90 (BAD95475.1), Human enterovirus 71 (CAL36654.1), Echovirus 1 strain Farouk (AAC63944.2), Human coxsackievirus B2 (AAD19874.1), Human enterovirus 86 (AAX47040.1), Human coxsackievirus B5 (AAF21971.1), Human echovirus 29 (AAQ73089.1), Human enterovirus 68 (AAR98503.1), Human enterovirus 70 (BAA18891.1), Bovine enterovirus (NP_045756.1), Porcine enterovirus A (NP_653145.1), Porcine enterovirus B (NP_758520.1), Simian enterovirus A (NP_653149.1), Human rhinovirus A (ABF51203.1), Human rhinovirus B (NP_041009.1).

Picobirnavirus trees: Human picobirnavirus strain 1-CHN-97 (AF246939.1), Human picobirnavirus strain 4-GA-91 (AF246940.1), Human picobirnavirus strain Hy005102 (NC_007027.1), Human picobirnavirus strain 2-GA-91 (AF245701.1), Human picobirnavirus strain 1-GA-91 (AF246612.1), Porcine picobirnavirus 2 (EU104360.1).

Anellovirus trees: TGP96 Torque teno virus (AB041962), Pt-TTV8-II Torque teno virus (AB041963), CBD231 TTV-like mini virus (AB026930), Mf-TTV9 Torque teno virus (AB041959), Mf-TTV3 Torque teno virus (AB041958), KC009/G4 Torque teno virus (AB038621), TA278/G1 Torque teno virus (AB008394), Pt-TTV6 Torque teno virus (AB041957), TUS01/G3 Torque teno virus (AB017613), PMV/G2 Torque teno virus (AF261761), JT33F/G5 Torque teno virus (AB064606), MD1-073 Torque teno midi virus (AB290918), MD2-013Torque teno midi virus (AB290919), Tbc-TTV14 Torque teno virus (AB057358), Sd-TTV31 Torque teno virus (AB076001), Fc-TTV4 Torque teno virus (AB076003), Cf-TTV10 Torque teno virus (AB076002), So-TTV2 Torque teno virus (AB041960), At-TTV3 Torque teno virus (AB041961).

Calicivirus trees: Camberwell (AAD33960.1), MD-2004 (ABG49508.1), Carlow(ABD73935.1), Snow Mountain virus (AAN08111.1), Mc37 (AAS47823.1), Hawaii(AAB97767.2), Norwalk(AAB50465.1), Southampton (AAA92983.1), Chiba(BAB18266.1), Hesse(AAC64602.1), BoJena-DEU-98 (CAA09480.1), Murine (AAO63098.2), SU17(BAC11827.1), Dumfries (AAM95184.2), SU25-JPN(BAC11830.1), SU1-JPN(BAC11815.1), Desert Shield (AAA16284.1), Melksham (CAA57461.1), Toronto-24 (AAA18929.1), Sw918 (BAB83515.1), OH-QW101 (AAX32876.1).

Endonuclease-like sequences for D03 tree (mimvirus-like sequences): *Bacteroides caccae* (ZP_01959575.1), *Acanthamoeba* mimivirus (YP_142599.1), *Eubacterium dolichum* (ZP_02077753.1), *Staphylococcus* phage K (YP_024462.1), *Lactobacillus* phage LP65 (YP_164778.1), *Lactococcus* phage bIL170 (NP_047162.1), *Lactococcus* phage r1t (NP_695069.1), *Burkholderia vietnamiensis* G4 (YP_001119011.1), *Streptococcus pyogenes* (NP_607538.1), *Tetrahymena thermophila* (XP_001029162.1), *Bacteroides vulgatus* (YP_001300673.1)

## Supporting Information

Figure S1 Composite analysis of all sequences. Sequences from all 12 libraries were categorized based on the best tBLASTX scores (E-value: <1e-5) as viral, phage, bacterial, human, fungal, other, or unassigned. Numbers in parenthesis represent the number of sequences in each category.
(0.21 MB TIF)

Figure S2 Phylogenetic analysis of picobirnavirus-like sequence reads. Phylogenetic trees were generated by comparing the translated amino acid sequence of individual sequence reads to members of the unclassified taxa picobirnavirus. The trees were created using the maximum parsimony method with 1,000 replicates. Bootstrap values over 700 are shown.
(0.43 MB TIF)

Figure S3 Phylogenetic analysis of Picornaviridae-like sequence reads. Phylogenetic trees were generated by comparing the translated amino acid sequence of individual sequence reads to members of the Picornaviridae family. The trees were created using the maximum parsimony method with 1,000 replicates. Bootstrap values over 700 are shown. CVA = Coxsakievirus A, CVB = Coxsackievirus B, BEV = Bovine Enterovirus, EV = Enterovirus, HRVA = Human Rhinovirus A, HRVB = Human Rhinovirus B, PEV = Porcine Enterovirus, PV = Poliovirus, SEVA = Simian Enterovirus A.
(0.66 MB TIF)

Figure S4 Phylogenetic analysis of anellovirus-like sequence reads. Phylogenetic trees were generated by comparing the translated amino acid sequence of individual sequence reads to anelloviruses. The trees were created using the maximum parsimony method with 1,000 replicates. Bootstrap values over 700 are shown.
(0.37 MB TIF)

Figure S5 Phylogenetic analysis of Caliciviridae-like sequence reads. Phylogenetic trees were generated by comparing the translated amino acid sequence of individual sequence reads to the A) NS4 (3A-like) protein or B) NS7 (RNAP) protein of caliciviruses. The trees were created using the maximum parsimony method with 1,000 replicates. Bootstrap values over 700 are shown.
(0.58 MB TIF)

Figure S6 Phylogenetic analysis of endonuclease-like sequence reads. Phylogenetic trees were generated by comparing the translated amino acid sequence of two individual sequence reads to endonuclease sequences derived from mimivirus, phage, and bacterial species representing some of the top scoring BLAST hits. The trees were created using the maximum parsimony method with 1,000 replicates. Bootstrap values over 700 are shown.
(0.54 MB TIF)
