# Supplementary figures and images for: Metagenomic Analysis of Human Diarrhea: Viral Detection and Discovery
Source: PLoS Pathog. 2008 Feb 29;4(2):e1000011. doi: 10.1371/journal.ppat.1000011 (PMC2290972; doi:10.1371/journal.ppat.1000011)

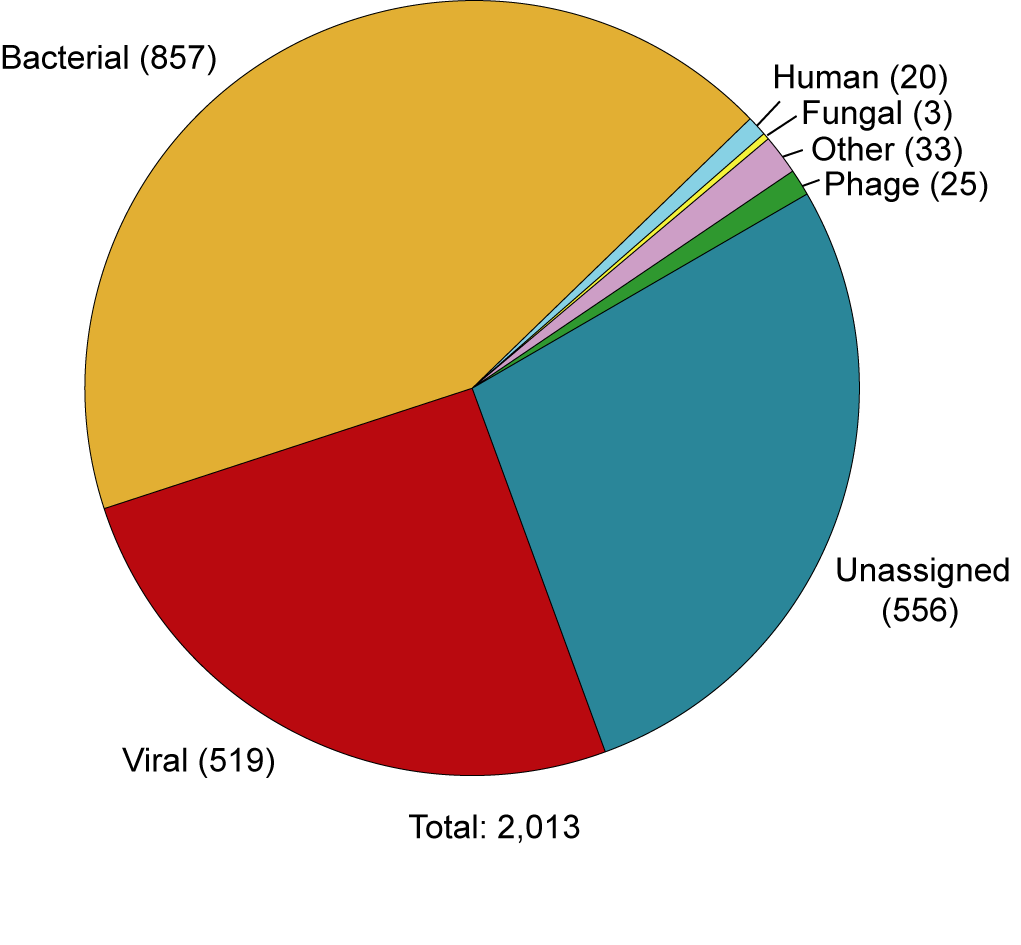

Supplement: Figure S1 — Composite analysis of all sequences. Sequences from all 12 libraries were categorized based on the best tBLASTX scores (E-value: <1e-5) as viral, phage, bacterial, human, fungal, other, or unassigned. Numbers in parenthesis represent the number of sequences in each category. (0.21 MB TIF) [file ppat.1000011.s001.tif]

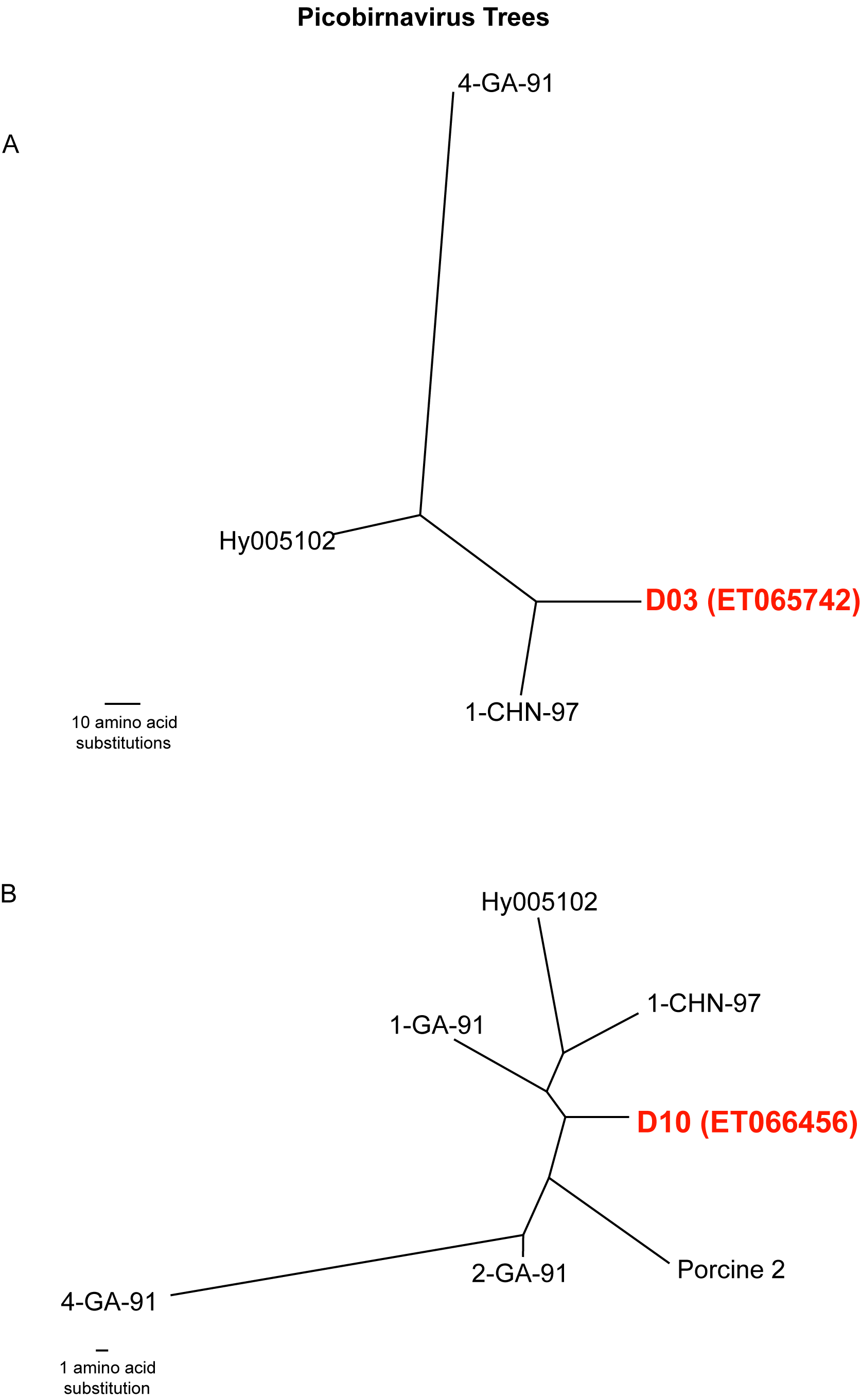

Supplement: Figure S2 — Phylogenetic analysis of picobirnavirus-like sequence reads. Phylogenetic trees were generated by comparing the translated amino acid sequence of individual sequence reads to members of the unclassified taxa picobirnavirus. The trees were created using the maximum parsimony method with 1,000 replicates. Bootstrap values over 700 are shown. (0.43 MB TIF) [file ppat.1000011.s002.tif]

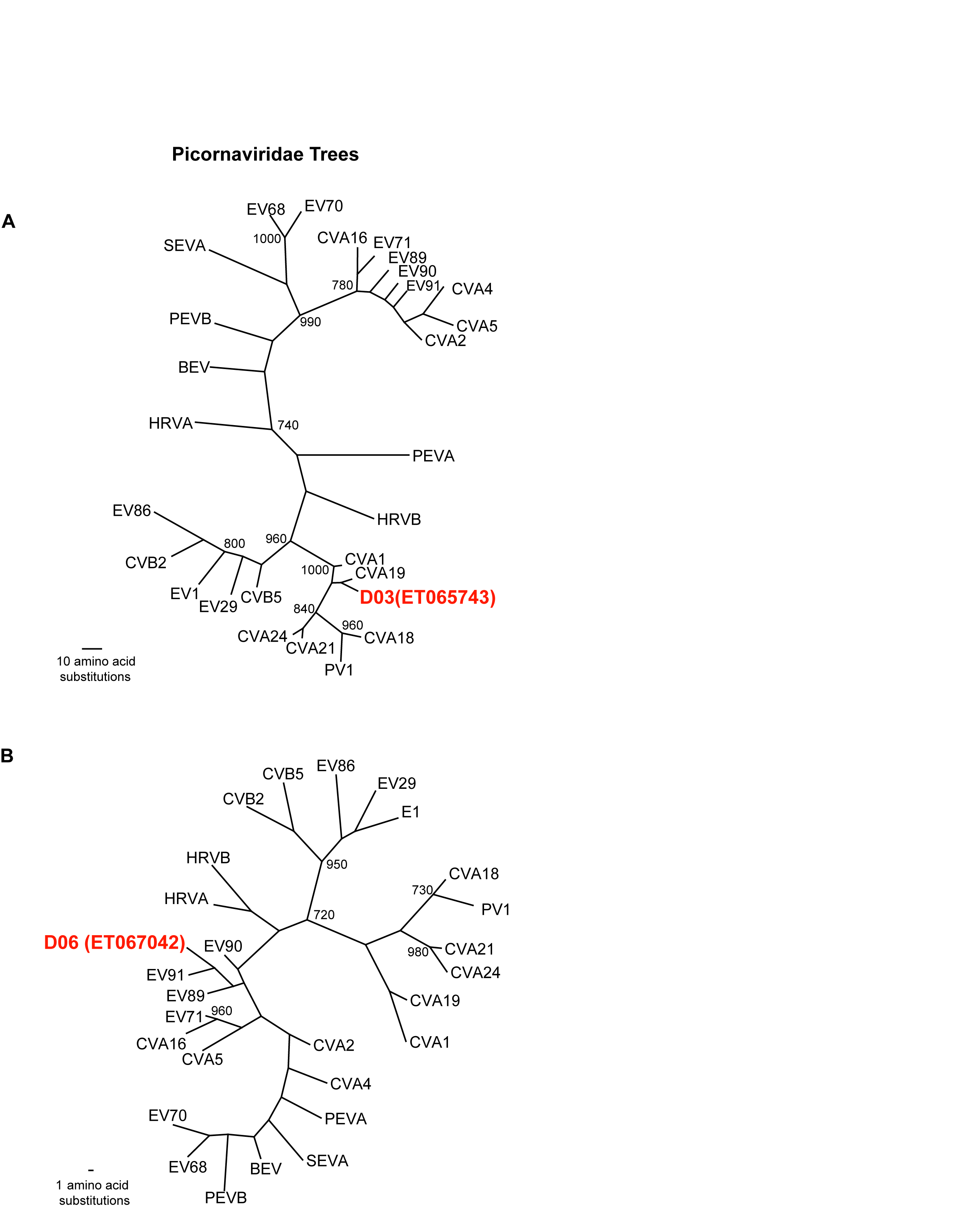

Supplement: Figure S3 — Phylogenetic analysis of Picornaviridae-like sequence reads. Phylogenetic trees were generated by comparing the translated amino acid sequence of individual sequence reads to members of the Picornaviridae family. The trees were created using the maximum parsimony method with 1,000 replicates. Bootstrap values over 700 are shown. CVA = Coxsakievirus A, CVB = Coxsackievirus B, BEV = Bovine Enterovirus, EV = Enterovirus, HRVA = Human Rhinovirus A, HRVB = Human Rhinovirus B, PEV = Porcine Enterovirus, PV = Poliovirus, SEVA = Simian Enterovirus A. (0.66 MB TIF) [file ppat.1000011.s003.tif]

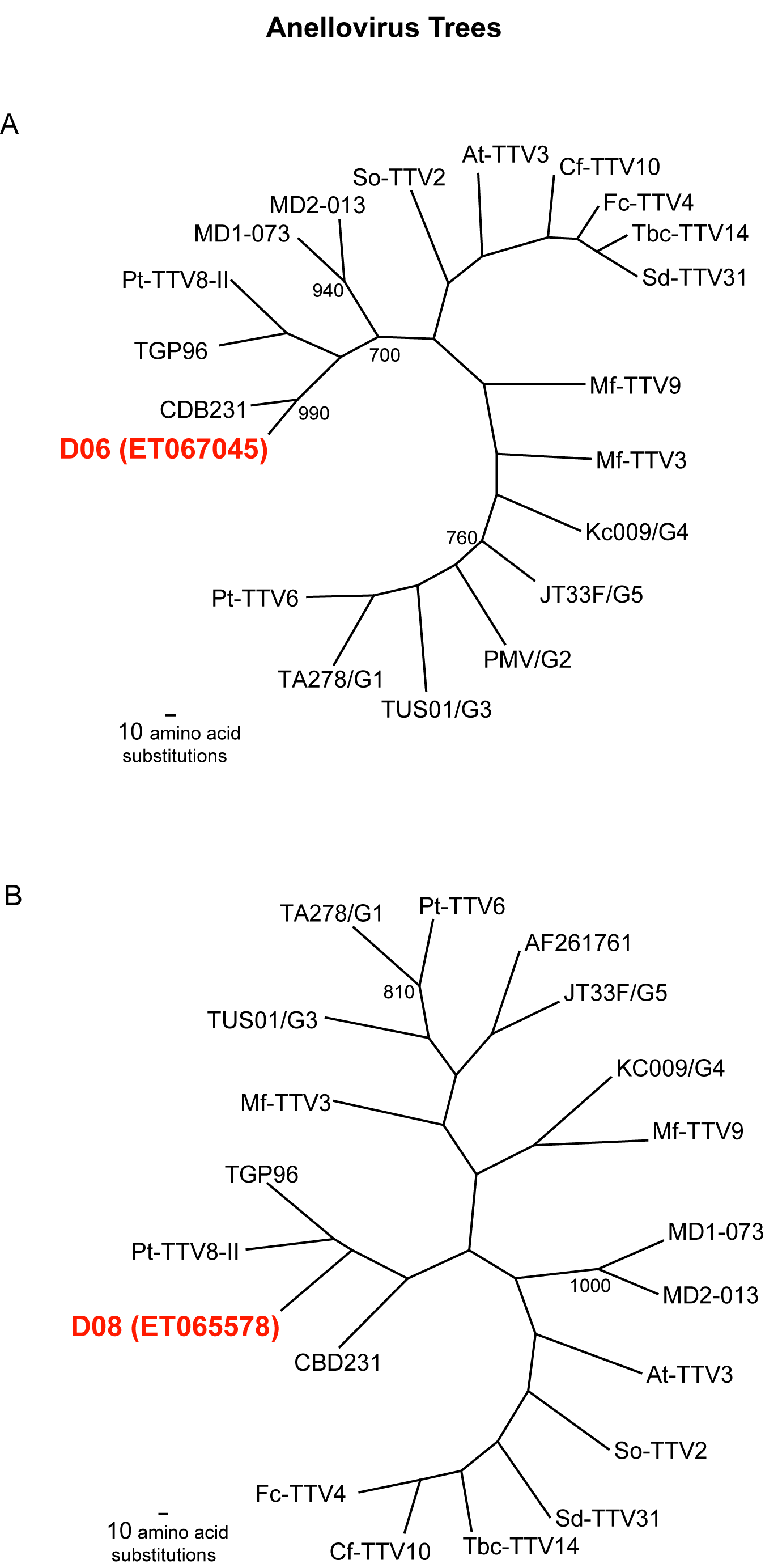

Supplement: Figure S4 — Phylogenetic analysis of anellovirus-like sequence reads. Phylogenetic trees were generated by comparing the translated amino acid sequence of individual sequence reads to anelloviruses. The trees were created using the maximum parsimony method with 1,000 replicates. Bootstrap values over 700 are shown. (0.37 MB TIF) [file ppat.1000011.s004.tif]

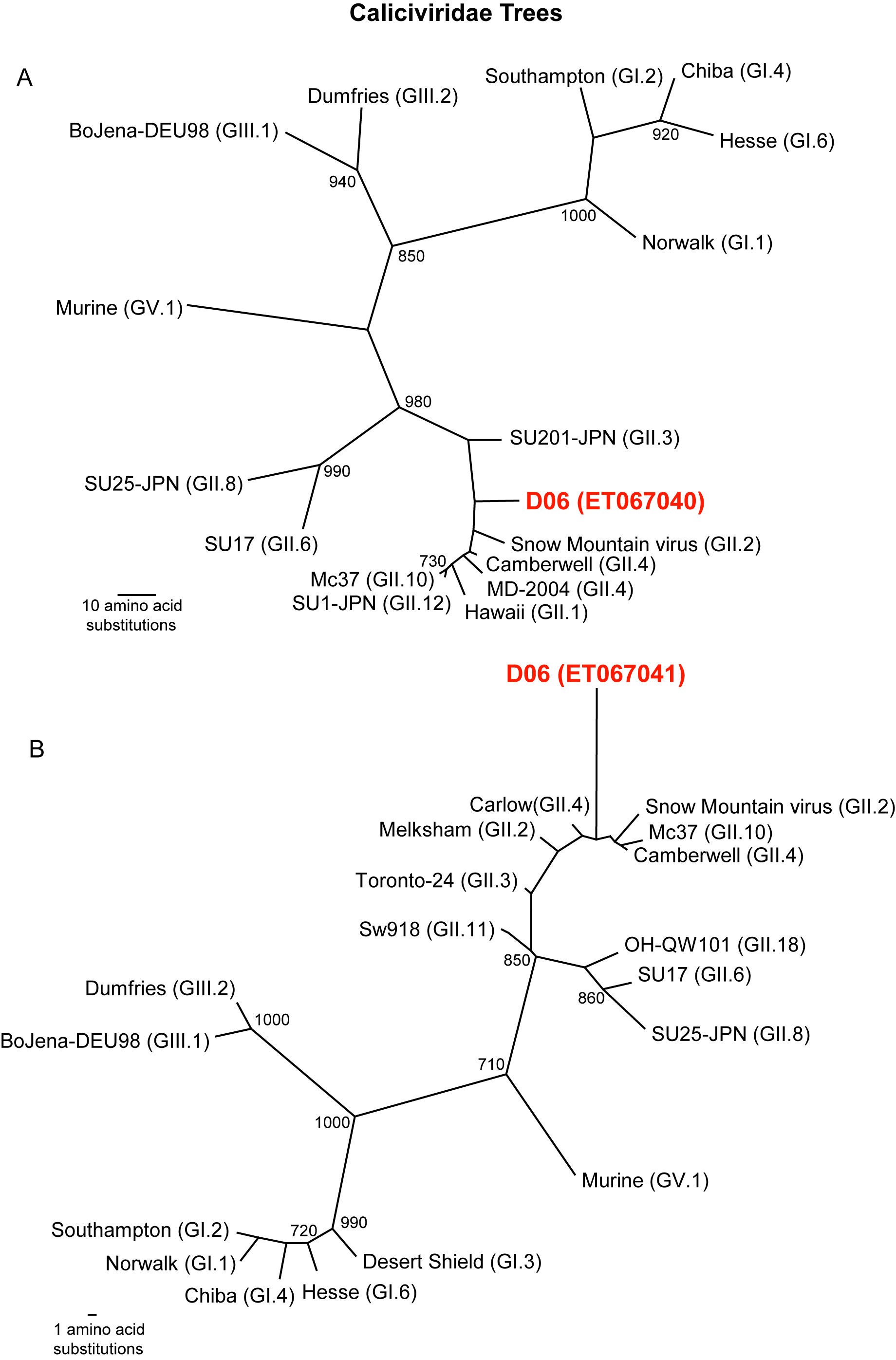

Supplement: Figure S5 — Phylogenetic analysis of Caliciviridae-like sequence reads. Phylogenetic trees were generated by comparing the translated amino acid sequence of individual sequence reads to the A) NS4 (3A-like) protein or B) NS7 (RNAP) protein of caliciviruses. The trees were created using the maximum parsimony method with 1,000 replicates. Bootstrap values over 700 are shown. (0.58 MB TIF) [file ppat.1000011.s005.tif]

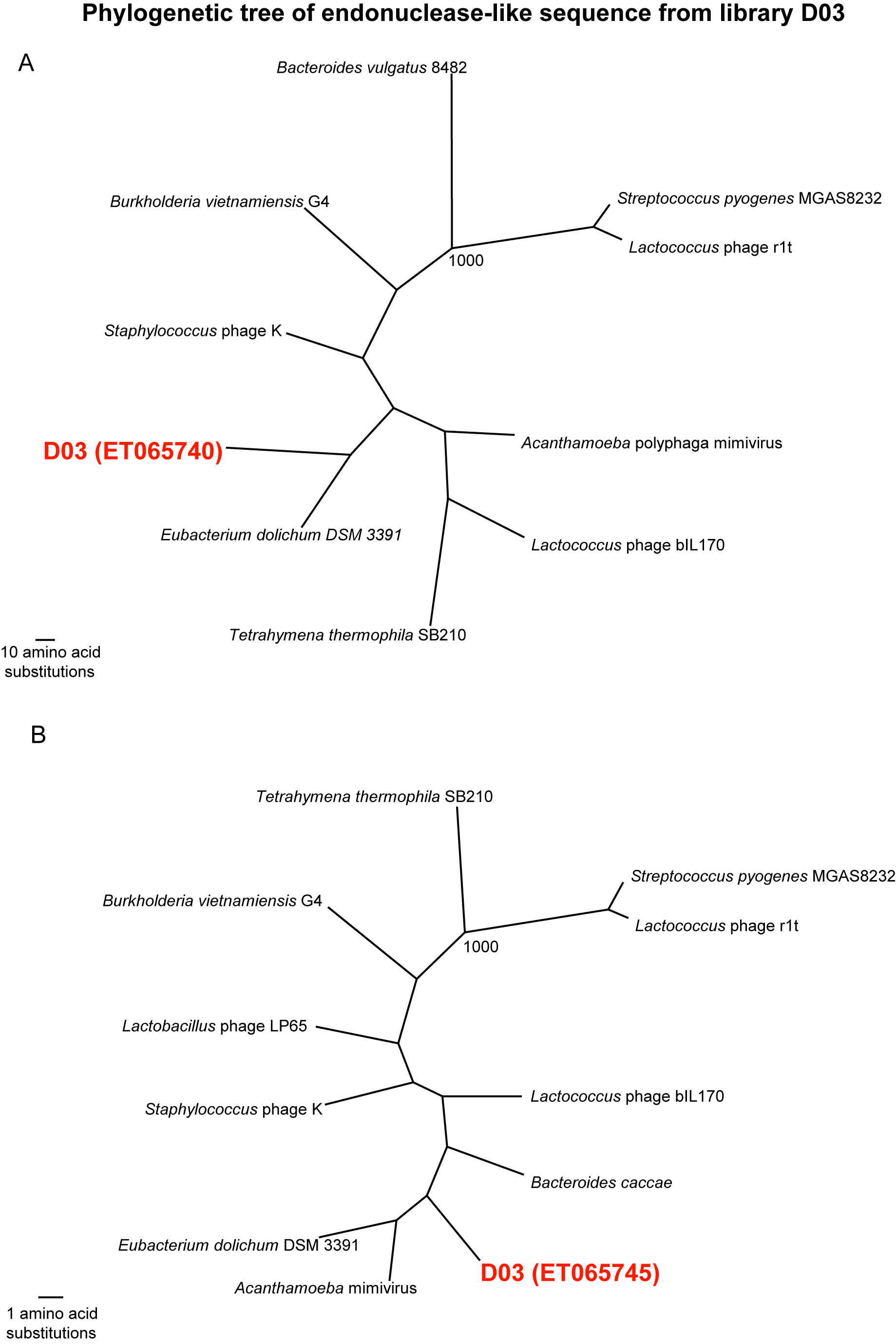

Supplement: Figure S6 — Phylogenetic analysis of endonuclease-like sequence reads. Phylogenetic trees were generated by comparing the translated amino acid sequence of two individual sequence reads to endonuclease sequences derived from mimivirus, phage, and bacterial species representing some of the top scoring BLAST hits. The trees were created using the maximum parsimony method with 1,000 replicates. Bootstrap values over 700 are shown. (0.54 MB TIF) [file ppat.1000011.s006.tif]
